# The Role of Integration Host Factor in Escherichia coli Persister Formation

**DOI:** 10.1128/mbio.03420-21

**Published:** 2022-01-04

**Authors:** Samantha E. Nicolau, Kim Lewis

**Affiliations:** a Antimicrobial Discovery Center, Department of Biology, Northeastern University, Boston, Massachusetts, USA; University of Oklahoma Health Sciences Center

**Keywords:** IHF, global regulator, metabolism, noise, persistence

## Abstract

Persisters represent a small subpopulation of cells that are tolerant of killing by antibiotics and are implicated in the recalcitrance of chronic infections to antibiotic therapy. One general theme has emerged regarding persisters formed by different bacterial species, namely, a state of relative dormancy characterized by diminished activity of antibiotic targets. Within this framework, a number of studies have linked persister formation to stochastic decreases in energy-generating components, leading to low ATP and target activity. In this study, we screen knockouts in the main global regulators of Escherichia coli for their effect on persisters. A knockout in integration host factor (IHF) had elevated ATP and a diminished level of persisters. This was accompanied by an overexpression of isocitrate dehydrogenase (Icd) and a downregulation of isocitrate lyase (AceA), two genes located at the bifurcation between the tricarboxylic acid (TCA) cycle and the glyoxylate bypass. Using a translational *ihfA-mVenus* fusion, we sort out rare bright cells, and this subpopulation is enriched in persisters. Our results suggest that noise in the expression of *ihf* produces rare cells with low Icd/high AceA, diverting substrates into the glyoxylate bypass, which decreases ATP, leading to antibiotic-tolerant persisters. We further examine noise in a simple model, the *lac* operon, and show that a knockout of the *lacI* repressor increases expression of the operon and decreases persister formation. Our results suggest that noise quenching by overexpression serves as a general approach to determine the nature of persister genes in a variety of bacterial species and conditions.

## INTRODUCTION

Two different types of mechanisms allow bacteria to evade killing by antibiotics: genetically encoded resistance (1) and phenotypic tolerance conferred by persister cells (2). Several lines of evidence point to persisters as a major culprit of drug-tolerant infections. Most chronic infections, such as those associated with biofilms, are actually caused by pathogens that are susceptible to antibiotics. Biofilms protect pathogens from the large components of the immune system, and the persister cells they harbor survive antibiotic treatment (3). The result is a relapsing chronic infection. Treatment of a biofilm infection of Staphylococcus aureus in a mouse model with regular antibiotics leaves a substantial population of live bacteria (4). These can be eliminated by ADEP4, a dysregulator of the Clp protease (4, 5), or with a membrane-acting retinoid compound (6). Further linking persisters to disease are findings of high-persister mutants among clinical isolates from patients with chronic infections caused by Pseudomonas aeruginosa, Mycobacterium tuberculosis, and Escherichia coli (7–10). Notably, the host environment favors a transition into a drug-tolerant state. For example, entrance of Salmonella into macrophages induces persister formation (11), and a general shift into a nonreplicating, drug-tolerant state is observed in M. tuberculosis residing in hypoxic granulomas (12–15). Apart from recalcitrance of chronic infections, additional significance of tolerance lies in its link to antibiotic resistance. Selection for increased tolerance can favor subsequent development of resistance in E. coli and S. aureus (16–18). This relationship was not observed in P. aeruginosa from patients with cystic fibrosis (10) and is likely dependent on the species of pathogen and its growth conditions. However, in all cases of chronic infection, a large lingering population of pathogen is fertile ground for the acquisition of resistance (19).

Given the importance of persisters in infectious diseases, there has been considerable interest in understanding the mechanism of their formation and the linked problem of antibiotic tolerance. Since bactericidal antibiotics act by corrupting their targets, we proposed that target inactivity in persisters is the mechanistic basis of tolerance (20, 21). In E. coli, which has served as a model for persister studies, there is at least one case for which there is good understanding of the mechanism of target shutdown. In the presence of fluoroquinolones that damage DNA, SOS response is activated and turns on expression of the TisB toxin. TisB is an endogenous antimicrobial peptide that forms a membrane channel (22), leading to a decrease in proton-motive force, ATP, target shutdown, and multidrug tolerance (22–24). The action of TisB can be replicated by artificially applying an uncoupler, CCCP (23), or depleting ATP with arsenate (25, 26); in both cases, this produces tolerance of antibiotics. These experiments show that low energy/low ATP can be causal to persister formation.

In cells not expressing TisB, noise in the expression of energy-generating components could lead to persisters. Indeed, sorting on the basis of GFP translational fusions showed that the dim population, cells with low levels of tricarboxylic acid (TCA) enzymes, was enriched in persisters (27, 28).

In this study, we sought to investigate the role of global regulators in persister formation in E. coli. Global regulators control multiple functions, and noise in their gene expression may result in low expression of downstream persister genes, leading to persister formation. In E. coli a handful of transcriptional regulators directly control expression of 51% of genes (29). These regulators include FNR, IHF, FIS, ArcA, NarL, and Lrp (30, 31). FNR senses oxygen levels (32–34), while ArcA is part of a two-component system (TCS) that senses the redox state of the respiratory chain (35). These regulators are most important under anaerobic conditions and regulate genes involved in the transition from aerobic to anaerobic growth (36). IHF, HNS, and FIS are all structural proteins that respond specifically to the level of DNA supercoiling, which is a reflection of the overall energy state of the cell (37–41). NarL, part of another TCS, senses nitrate levels (42–45), while Lrp senses l-leucine levels in the cell (46–48).

Several global regulators had been previously linked to persister formation (49–52). In this study, we extend the examination of global regulators with a focus on the control of energy-producing components. We show that IHF controls the expression of isocitrate lyase, the first step in the glyoxylate bypass, as well as isocitrate dehydrogenase, a key enzyme in the TCA cycle, and noise in IHF leads to persister formation.

## RESULTS

To identify global regulators involved in persister formation, we examined survival of E. coli knockout strains challenged with antibiotic. Single-gene deletions of global regulators *narL*, *lrp*, *ihfA*, *ihfB*, *fnr*, *arcA*, *arcB*, *fis*, or *hns* from the Keio collection (53) were moved into the MG1655 background via P1 phage transduction. Strains were grown to stationary phase and challenged with a high dose of ciprofloxacin. The level of persisters strongly depends on the density of a growing culture, reaching a maximum at stationary phase of growth (54). We therefore chose to examine survival at stationary phase for a more reliable comparison among the various knockout strains. Stationary-phase cultures were challenged with a lethal dose of ciprofloxacin (10 μg/ml) that kills regular cells (23), and after 24 h, surviving persisters were enumerated by colony count.

**FIG 1:**
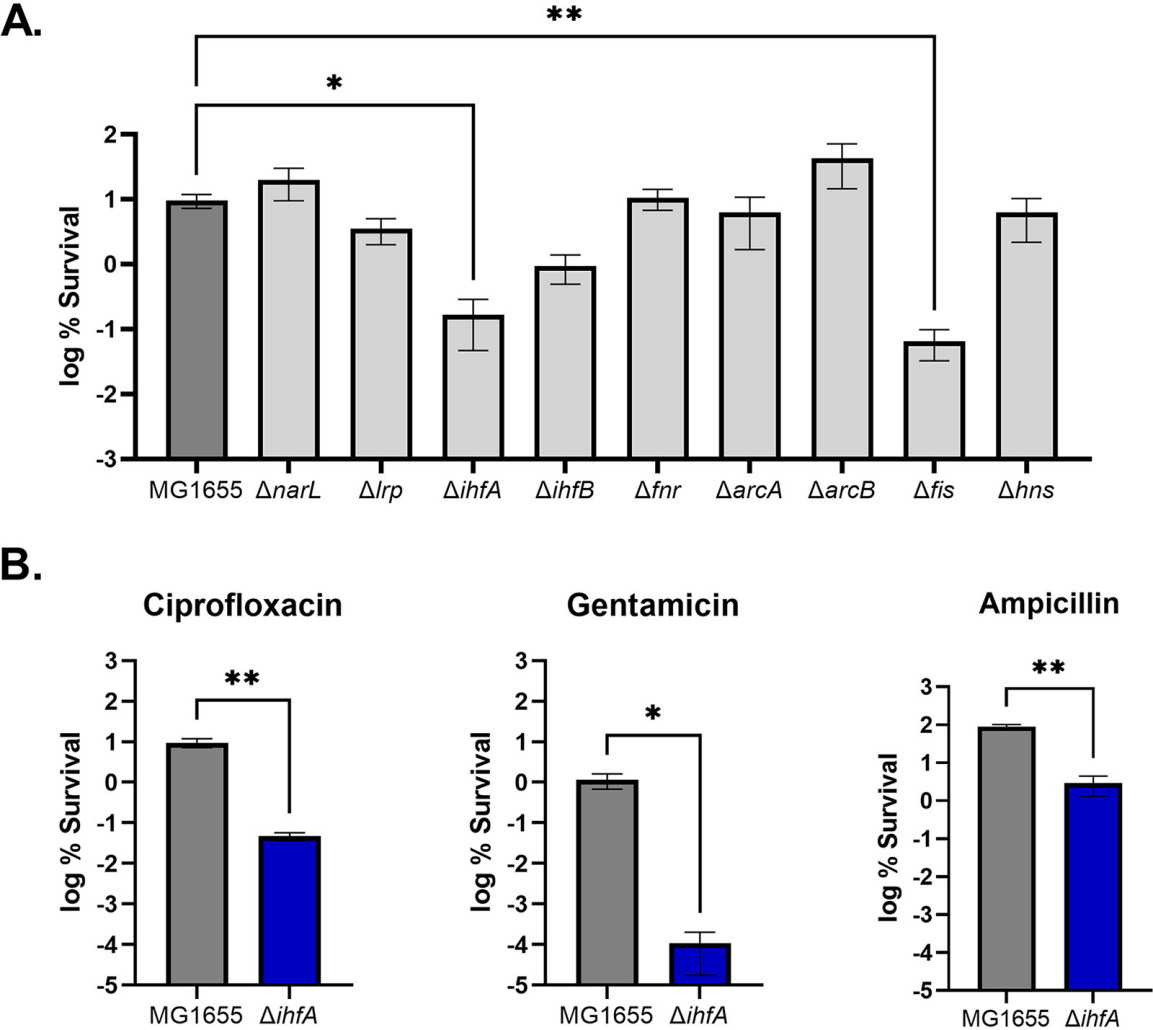
Effect of global regulators on cell survival. (A) Survival of global regulator knockouts challenged with ciprofloxacin. E. coli was grown to stationary phase in LB broth (LBB). Ciprofloxacin was added at 10 μg/ml and, after 24 h, plated for CFU. Δ*ihfA* (*P* < 0.05) and Δ*fis* (*P* < 0.01) strains were found to have a significant difference in survival. Significance in survival among strains was determined by the Kruskal-Wallis multiple-comparison test. (B) Survival of wild-type and Δ*ihfA* strains challenged with different antibiotics. Cultures were grown to stationary phase, challenged with ciprofloxacin at 10 μg/ml, gentamicin at 50 μg/ml, or ampicillin at 100 μg/ml, and, after 24 h, plated to enumerate CFU. The Δ*ihfA* strain had a significant decrease in percent survival compared to the wild type for all classes of antibiotics, as determined by a Mann-Whitney test (*P* < 0.05). Represented are the averages from two independent experiments performed in biological triplicate. Error bars represent standard errors of the means (SEM).

Of the mutants tested, a deletion in *ihfA* (and, to a lesser extent, *ihfB*) coding for the alpha subunit of integration host factor (IHF) and an *fis* knockout had significantly decreased survival after treatment with ciprofloxacin (Fig. 1A). An FIS deletion was previously identified as having a decreased persister level in Salmonella (51). It was reported that cells lacking *fis* had decreased survival compared to the wild type and increased expression of several genes associated with glutamate transport. The *fis* deletion strain was shown to have increased intracellular glutamate levels compared to the wild type, and when the promoter region of the glutamate transporter operon was deleted in a Δ*fis* background, the resulting strain had increased survival. It was therefore proposed that *fis* modulates persister levels through regulation of glutamate transport in Salmonella (51). IHF had been previously reported to have an impact on persister levels (49, 52), but the mechanism by which it affects tolerance is unknown. We therefore sought to study the link between IHF and persisters. First, we tested additional antibiotics and found that the Δ*ihfA* strain had decreased survival in the presence of gentamicin and ampicillin as well (Fig. 1B).

**FIG 2:**
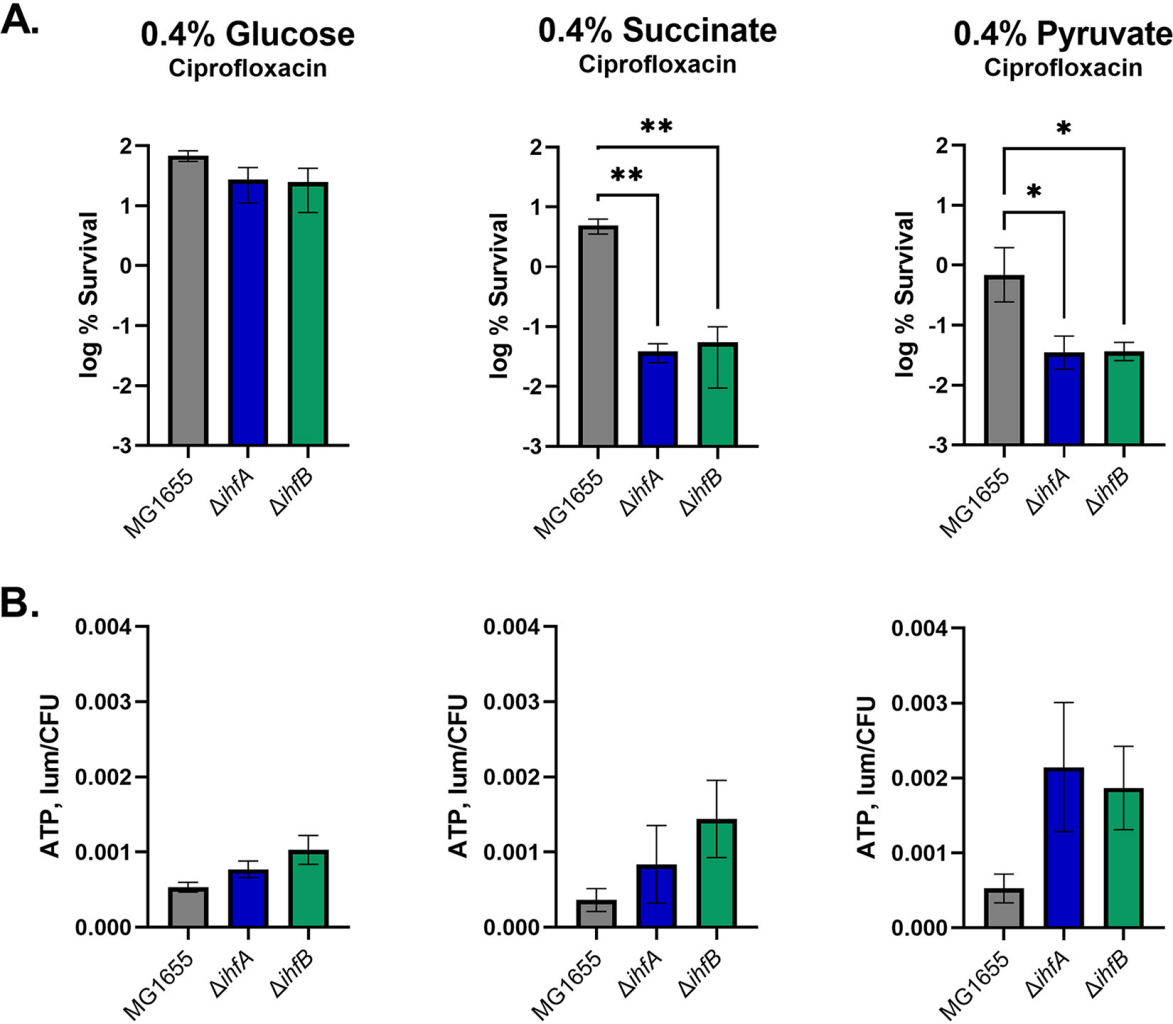
Persister levels of *ihf* mutants correlate with ATP. Strains were grown to stationary phase in minimal medium with different carbon sources. (A) Effects of *ihf* deletions on survival. Stationary-phase cultures were challenged with ciprofloxacin at 10 μg/ml. Persister levels were determined by plating and colony counting. *ΔihfA* and *ΔihfB* strains had significantly decreased survival compared to the wild type when grown on succinate (*P* < 0.005) or pyruvate (*P* < 0.05). Statistical significance between the wild type and mutants was determined using analysis of variance (ANOVA) followed by Sidak’s multiple-comparison test. (B) ATP levels of *ihf* deletion strains. ATP was measured in the bulk of the population using the BacTiter-Glo luciferase assay. This figure represents the average from two independent experiments performed in biological triplicate. Error bars represent SEM.

To simplify analysis, the *ihfA* and *ihfB* mutants were retested in defined minimal media with either glucose, succinate, or pyruvate as the single carbon source. These carbon sources were chosen as they enter central metabolism at three different points, glycolysis, late TCA cycle, and early TCA cycle, respectively. When grown on glucose, there was no difference in survival between the wild type and mutants; however, in media with succinate or pyruvate, the deletion mutants had a decreased level of persisters, comparable to that of cultures grown in the LB broth (LBB) medium (Fig. 2A). It has been reported that persisters have decreased ATP levels (25, 26); therefore, we determined the ATP levels in stationary-phase cells of the mutants using the BacTiter-Glo luciferase assay (Fig. 2B). In general, there was an increase in ATP level in the *ihfA* and *ihfB* deletion mutants. ATP levels had an opposite correlation with survival, and an increase in ATP was most pronounced in media with succinate and pyruvate. Higher levels of ATP support the activity of targets, diminishing tolerance to antibiotics (26). Our results therefore are in good agreement with previous studies showing an inverse relationship between ATP and persister levels.

**FIG 3:**
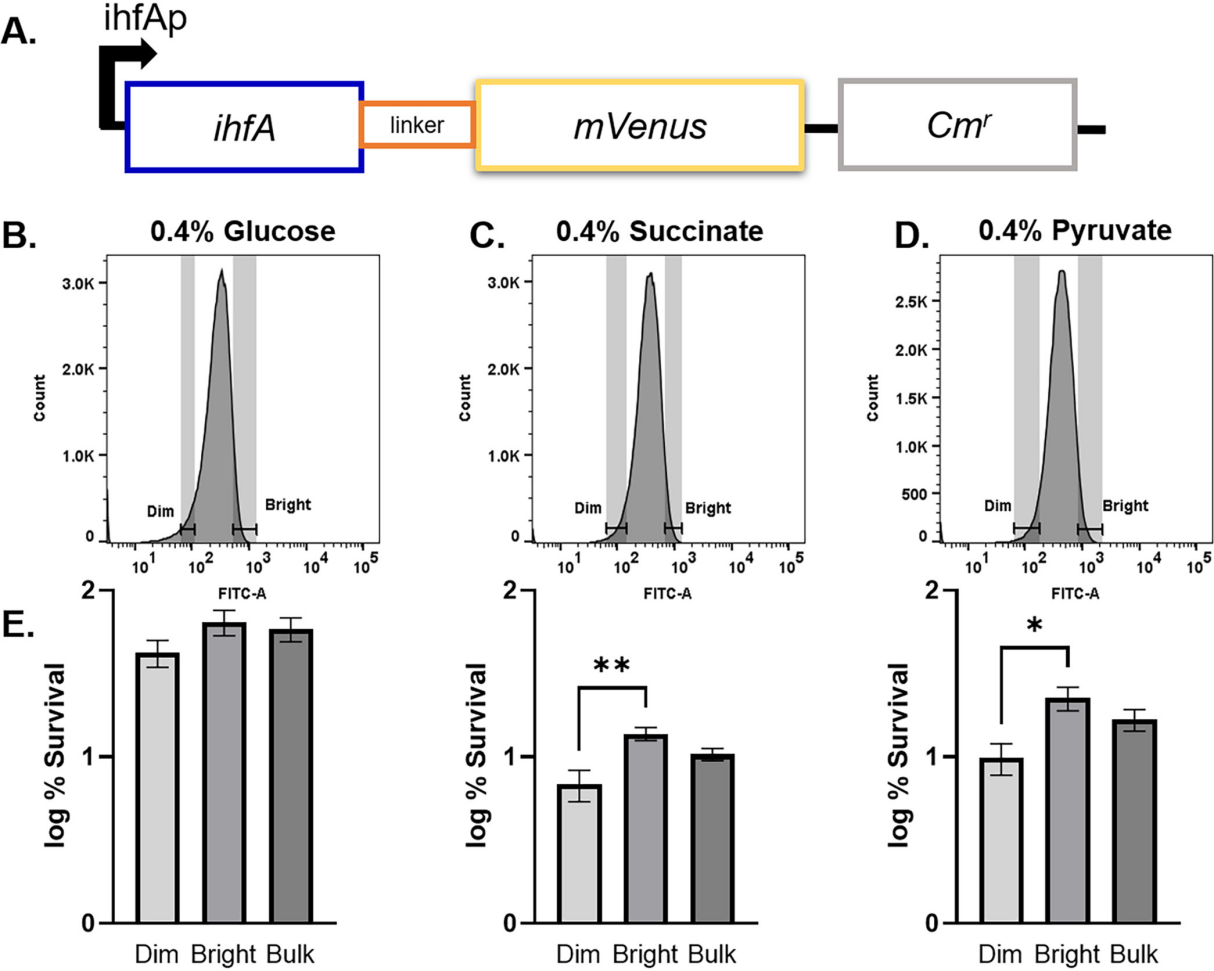
Sorting of cells with high and low expression levels of IhfA-mVenus. (A) Schematic of *ihfA-mVenus* translational fusion. (B to D) Fluorescence distribution of *ihfA-mVenus* reporter in medium with various carbon sources. (E) Percent survival of the dim and bright fractions as well as the bulk of the culture. A significant decrease in survival was seen in the dim fraction compared to the bright fraction when the reporter was grown on succinate (*P* < 0.005) or pyruvate (*P* < 0.05). Statistical significance was determined using analysis of variance (ANOVA) followed by Sidak’s multiple-comparison test. Bars represent averages from two independent experiments performed in biological triplicate. Error bars represent SEM.

In a medium with succinate or pyruvate, the level of persisters drops 20- to 100-fold in *ihf* mutants, showing that formation of the majority of antibiotic-tolerant cells under these conditions is controlled by the IHF global regulator. Persisters in the wild type under the same conditions constitute approximately 5% of the total population (Fig. 2A), suggesting that they form stochastically. We reasoned that noise in the expression of *ihf* may be responsible for the formation of the persister subpopulation. In order to test this, we constructed an *ihf* expression reporter that could be used to isolate individual cells with different levels of IHF. A chromosomally encoded *ihfA-mVenus* translational fusion was constructed for this purpose (Fig. 3A). The reporter strain was grown to stationary phase on each of the three carbon sources tested previously, glucose, succinate, and pyruvate, and challenged with ciprofloxacin. After 24 h of treatment, cells were analyzed by fluorescence-activated cell sorting (FACS). There is variation in the expression level of IHF in all media, showing considerable noise in the expression of the IhfA protein, which will enable sorting of dim and bright populations (Fig. 3B to D). The distribution was not significantly different before and after antibiotic treatment (data not shown). To isolate cells with low and high levels of expression of IhfA, dim and bright cells were gated (Fig. 3B to D) and sorted onto agar plates. In a culture grown on glucose, there was not an apparent enrichment for persisters in either fraction sorted. When *ihfA-mVenus* was grown on succinate or pyruvate, cells expressing low levels of *ihfA* had significantly decreased survival compared to the bright cells as well as the bulk of the population (Fig. 3E). These single-cell data are consistent with results obtained with a bulk culture, where there was no change in survival in glucose but a decrease in survival of the *ihf* deletion mutants in succinate and pyruvate. Not surprisingly, deletion produces a stronger decrease in persisters than cells with low levels of expression of *ihf*, but in both cases, the global regulator appears to be the main determinant of persister formation.

**FIG 4:**
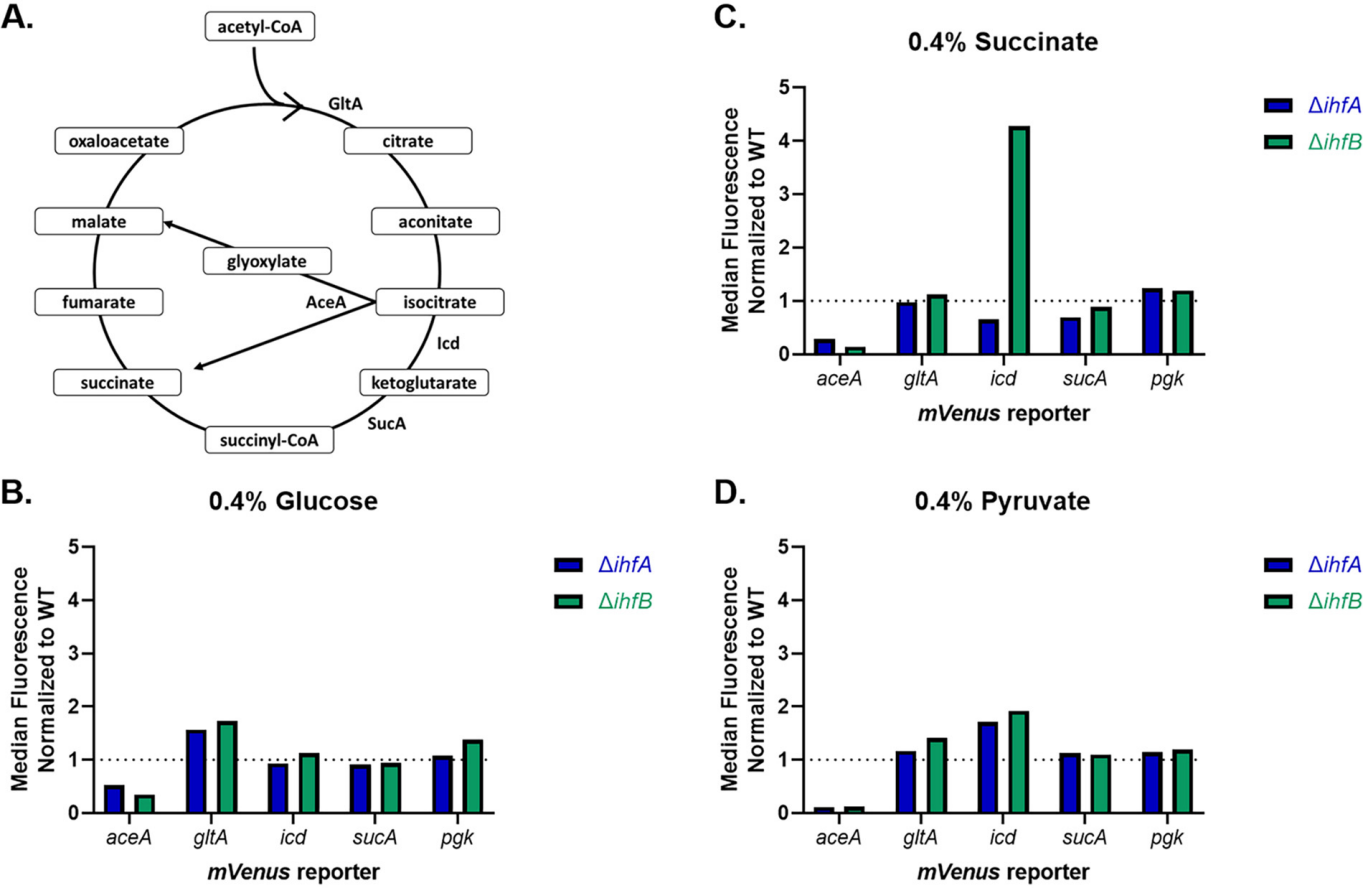
Expression of TCA cycle enzymes in wild-type and IHF mutant backgrounds. (A) Schematic of the TCA cycle. Translational fusions of key TCA cycle genes were moved into wild-type, Δ*ihfA*, or Δ*ihfB* background using P1 transduction. Fluorescence intensity from 100,000 events was collected for each strain in media containing glucose (B), succinate (C), or pyruvate (D). Median fluorescence intensity was calculated, normalized to the reporter in the wild-type background for all medium conditions and reporters, and graphed.

Given that an *ihfA* mutant showed a persister phenotype in media with succinate or pyruvate, we reasoned that the TCA cycle is involved in IhfA-dependent regulation of persister formation. According to RegulonDB (55), a number of TCA cycle genes are under the control of IHF: citrate synthase (*gltA*), 2-oxoglutarate decarboxylase complex (*sucAB*), and succinyl-coenzyme A (CoA) synthetase (*sucCD*). These constitute early (*gltA*) and late (*sucABCD*) TCA cycle genes or genes involved in the glyoxylate bypass (*aceBAK*). We next examined whether expression of these genes was affected in the deletion mutants. For this, mVenus translational fusions of *aceA* (isocitrate lyase), *sucA*, and *gltA* were moved into wild-type, Δ*ihfA*, and Δ*ihfB* strains via phage transduction. In addition, *icd-mVenus* (isocitrate dehydrogenase) and *pgk-mVenus* (phosphoglycerate kinase) were included as controls for TCA cycle and glycolysis genes, respectively, that presumably are not regulated by IHF. Cells were grown to stationary phase in medium containing glucose, succinate, or pyruvate and analyzed by FACS. Median fluorescence intensity was determined and normalized to expression of each reporter in the wild-type background. Reporters for *pgk*, *gltA*, and *sucA* in the deletion *ihf* background displayed expression similar to that of wild-type cells; however, there was an increase in *icd* expression and a decrease in *aceA* expression in the deletion mutants (Fig. 4). The decrease in *aceA* expression is consistent with the known role of IHF as an activator of this operon (56). We are not aware of a dedicated study examining possible regulation of *icd* expression by IHF. Previous studies have also shown that increased expression of *icd* results in a decrease in persisters (28). Interestingly, these two enzymes are located at the branch point between the TCA cycle and the glyoxylate bypass. Our data show that IHF acts as a repressor of *icd* expression and activator of the *ace* glyoxylate bypass. Taken together, our results suggest that noise in the expression of *ihf* produces cells with increased levels of this global regulator, which may repress transcription of a key TCA cycle component, Icd, and simultaneously divert substrates to the glyoxylate bypass, producing persisters. Deletion of *ihf* causes a dramatic decrease in persisters, which is probably due to its dual effect, overexpression of *icd* and repression of glyoxylate bypass genes, which will result in increased flux through the TCA cycle.

**FIG 5:**
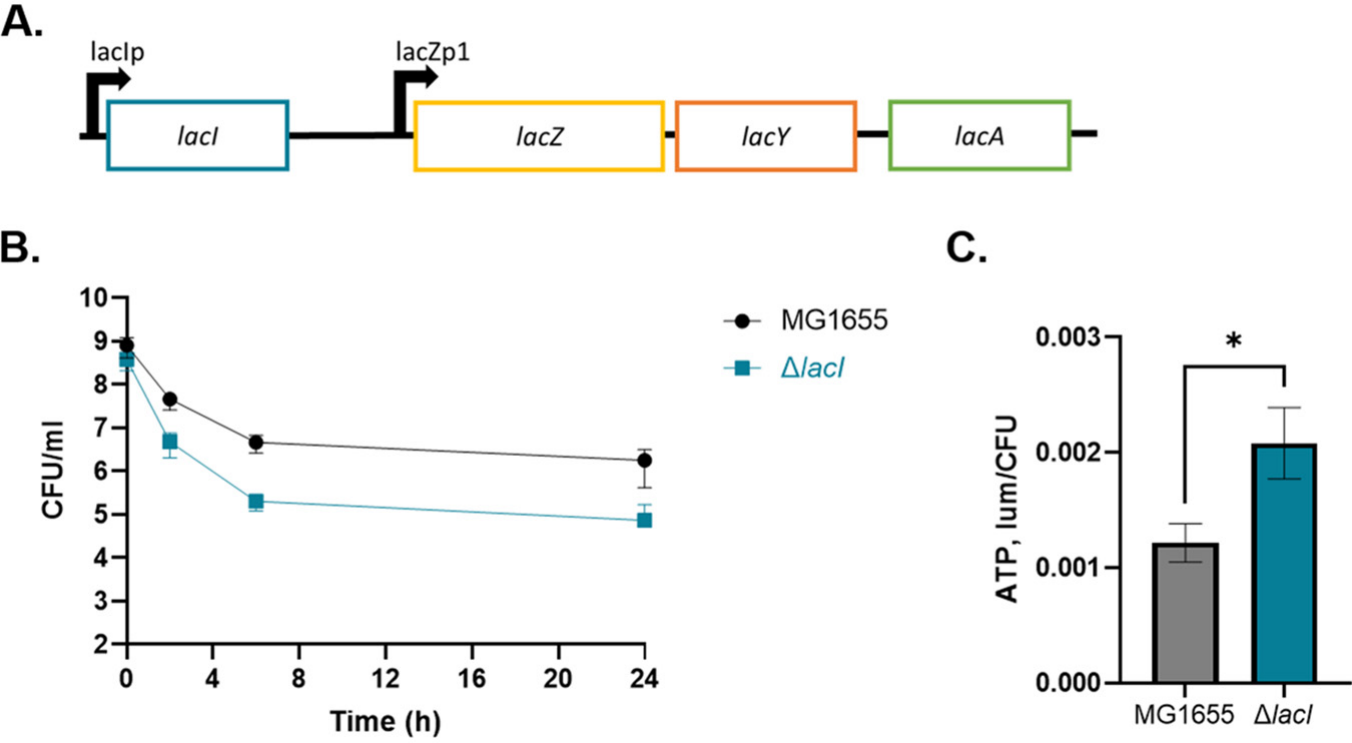
Effect of *lacI* deletion on persister formation. (A) Organization of the *lac* operon. LacI is the repressor of the lac operon. *lacZ* encodes beta-galactosidase, the enzyme responsible for cleaving lactose into glucose and galactose. *lacY* encodes the lactose permease, which imports lactose into the cell. *lacA* encodes the galactoside *O*-acetyltransferase. (B) Cultures were grown to stationary phase in M9 minimal medium supplemented with 1% l-lactose before treatment with ciprofloxacin. The *lacI* deletion had decreased persisters relative to the wild type. Represented are averages from six biological replicates with error bars quantifying standard deviations. (C) Relative ATP levels for wild-type and mutant strains. Cultures were grown to stationary phase, and ATP was measured in the bulk of the population using the BacTiter-Glo luciferase assay. The *lacI* deletion had significantly increased ATP compared to the wild-type strain (*P* < 0.05). Significance was determined using a Mann-Whitney test. Represented are averages from two independent experiments performed in biological triplicate. Error bars represent SEM.

IHF-governed regulation is highly complex but points to noise quenching, eliminating the noise in gene expression by overexpression, as a simple method to quantitatively determine the input of a gene into persister formation. We sought to examine whether noise quenching is indeed applicable to other genes. For this, we used what is probably the simplest model, the well-studied *lac* operon. The operon is regulated by the LacI repressor, which undergoes a conformational change when bound to allolactose, the inducer of the operon (57). This results in dissociation of LacI from the operator when lactose is present (58). The *lac* operon is particularly noisy, since the number of LacI proteins in the cell is low, 10 molecules on average (59). This favors substantial variation in cell-to-cell levels of expression. For example, even in the absence of lactose, there are rare individual cells that have no LacI and full expression of the operon (60, 61). We reasoned that deleting the *lacI* repressor will eliminate the noise in lactose catabolism by constitutively overexpressing the rest of the operon. As a result of this overexpression, cells will have increased lactose catabolism, which will lead to increased ATP and decreased persister levels when grown on lactose as the sole carbon source. We constructed an E. coli *lacI* deletion strain using allelic replacement (62). The deletion mutant and wild type were grown to stationary phase in the presence of lactose as the sole carbon source and assessed for persister and ATP levels. The *lacI* deletion had decreased persister levels compared to the wild type (Fig. 5B). ATP levels were determined using the luciferase assay, and *lacI* deletion was found to have increased ATP levels (Fig. 5C). This experiment suggests that the majority of persisters in wild-type E. coli under these conditions are rare cells with low levels of Lac proteins, leading to low ATP and antibiotic tolerance. Overexpression of the Lac proteins quenches the noise, decreasing the population of rare persister cells with low levels of these energy-generating components. This experiment also indicates that noise quenching by overexpression can be used as a general approach for identifying persister genes.

**FIG 6:**
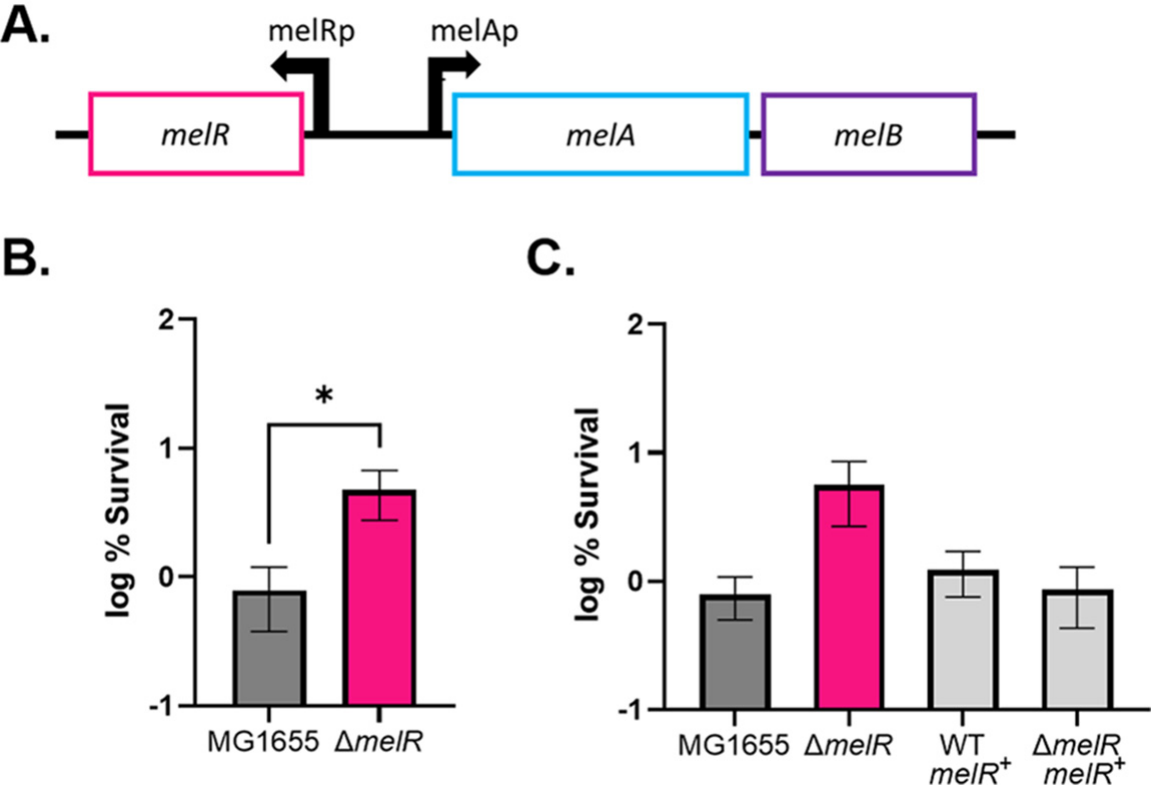
Noise quenching of the melibiose operon. (A) Organization of the melibiose operon. MelR is a transcriptional activator for the operon, which includes *melA*, an α-galactosidase, and *melB*, the melibiose transporter. (B) Δ*melR* strain survives significantly better than the wild type in stationary phase (*P* < 0.05). Cultures were grown to stationary phase in LBB plus 0.2% melibiose before challenge with ciprofloxacin (*n* = 9). Significance was determined using a Mann-Whitney test. Represented are averages from three independent experiments performed in biological triplicate. (C) Overexpression of *melR* rescues high survival phenotype. Cultures were grown to stationary phase in LBB plus 0.2% melibiose plus 0.1% l-arabinose (inducer) before challenge with ciprofloxacin. Represented are averages from two independent experiments performed in biological triplicate. Error bars represent SEM.

We next sought to apply this concept to additional operons. The melibiose operon was selected as it has an organizational structure similar to that of the *lac* operon (Fig. 6A) and, like LacI, MelR, the regulator of the operon, has low abundance, with fewer than 100 copies per cell (63). An important distinction is that MelR is a transcriptional activator, responsible for turning on expression of the operon rather than a repressor of the operon. We reasoned that deleting the *melR* activator will result in increased survival, as these cells will not be able to express the genes required for the import and breakdown of melibiose. A single deletion of *melR* was moved into the MG1655, wild-type background via P1 phage transduction from the Keio collection (53). The deletion and wild type were grown to stationary phase in LBB supplemented with melibiose, and persister level was assessed. The *melR* deletion had increased survival compared to the wild type (Fig. 6B). This result suggests that a deletion in *melR* leads to a decrease in expression of *melAB*, which results in persisters. To further elucidate the effect of noise in the *mel* operon, *melR* was cloned into pBAD33 (64). The resulting plasmid, pBAD33-*melR*, was transformed into the wild-type and Δ*melR* strains. Strains were grown overnight with inducer and assayed for survival against ciprofloxacin. Overexpression of *melR* in the wild-type and Δ*melR* backgrounds resulted in survival comparable to that of the wild-type strain carrying the empty vector (Fig. 6C) but did not decrease survival below this point. This experiment suggests that noise in expression of MelR is not sufficient to form persisters. This is why overexpression of the operon had no effect on the level of persisters: if noise is not prominent, there is nothing to quench. Apparently, noise in the downstream energy-producing pathway leads to persisters in this case. This is a cautionary tale showing that a persister phenotype observed in a deletion mutant cannot serve as a sole determinant for a persister gene.

## DISCUSSION

Our results identify IHF as an important regulator controlling persister formation in E. coli. IHF appears to repress isocitrate dehydrogenase (Icd), a key enzyme that lies at the branch point between the full TCA cycle and the glyoxylate bypass, and simultaneously activates expression of isocitrate lyase (AceA), the first enzyme of the bypass. This suggests that IHF redirects the carbon flow away from efficient energy production and into a pathway that is intended to conserve carbon. Typically, the glyoxylate bypass is activated when E. coli is grown on two-carbon substrates, such as acetate or fatty acids, to prevent the loss of carbon in the form of carbon dioxide. The cell will divert some of the carbon flux, at the isocitrate level, into the glyoxylate bypass to produce energy and four-carbon intermediates that can be used in other biosynthetic pathways (65). The flip side of this is that cells with lower levels of IHF will have a more active energy-producing metabolism. Indeed, deletion of *ihf* caused an increase in ATP and a concomitant decrease in persister level. These observations suggested that natural noise in the expression of IHF similarly affects tolerance. Indeed, sorting of bright cells carrying an *ihfA-mVenus* translational fusion were enriched in persisters. Notably, IHF has been described as one of the noisiest global regulators (66). An E. coli promoter fusion library was used to examine the noise associated with regulatory elements, and IHF had significantly higher levels of noise than the other sigma factors and transcriptional regulators assayed. Low levels of IHF will lead to increased expression of Icd and decreased expression of AceA, which will result in increased flux through the TCA cycle. Notably, IHF has also been shown to be necessary for efficient colonization of the bladder by uropathogenic E. coli, most likely due to defects in type 1 piliation (67). IHF appears to regulate both virulence factors and drug tolerance, highlighting its importance for infectious disease.

Noise quenching can serve as an approach to determine the involvement of a gene in persister formation, and we examined this in a simple model of the *lac* operon. The LacI repressor is present in only 10 copies on average in the cell, leading to considerable cell-cell variation. A mutant deleted in *lacI* showed a considerable decrease in the persister level, suggesting that noise quenching suppresses persister formation.

Candidate persister genes can be determined by a variety of approaches, such as analysis of knockout mutants or transcriptomes of isolated persisters. Knockouts, however, do not tell us whether natural noise in that gene is sufficient to produce rare cells with a persister phenotype. That requires additional investigation, such as single-cell analysis or noise quenching by overexpression.

The low-energy-level mechanism of persister formation our results point to is consistent with a large body of literature linking a metabolic downshift to antibiotic tolerance (2, 52, 68). Such downshift typically occurs in response to an external factor, such as starvation or hypoxia. For example, M. tuberculosis cells in a hypoxic granuloma enter into a nonreplicating drug-tolerant state. This population-wide shift is controlled by the DosR kinase that induces expression of the triacylglycerol synthase *tgs1*. Under hypoxia, *tgs1* redirects acetyl-CoA to the synthesis of storage triglycerides, away from the TCA cycle, contributing to a drop in ATP, antibiotic tolerance, and growth arrest (68). This switch resembles the IHF control of the carbon flux between the TCA cycle and the glyoxylate bypass, similarly affecting antibiotic tolerance. The immune system can also serve as an active inducer of persistence. As we already mentioned, entrance of Salmonella into macrophages causes a sharp increase in persisters (11); granuloma in tuberculosis is formed by immune cells (12–15). It was recently reported that reactive oxygen species produced by activated macrophages *in vitro* and *in vivo* inhibits the Fe-sulfur-containing aconitase of the TCA cycle and diminishes ATP of S. aureus, leading to antibiotic tolerance (69). Apart from the immune system, other pathogens may affect persister formation. In a mixed infection, 2-heptyl-4-hydroxyquinoline *N*-oxide (HQNO) produced by P. aeruginosa inhibits respiration of S. aureus, leading to drug tolerance (70).

Notably, in these population-wide metabolic downshifts, antibiotic tolerance is usually incomplete. For example, during DosR-controlled redirection of energy to triglyceride storage, ciprofloxacin kills 90% of M. tuberculosis cells, and a deletion in *tgs1* leads to a further ∼10-fold decrease in antibiotic tolerance. This means that the surviving Δ*tgs1* cells are persisters that are likely produced stochastically.

Not all persisters in a population growing in the absence of external stressors form through an energy-dependent mechanism. One well-established mechanism is based on the HipA toxin in E. coli. HipA is a protein kinase (71) that phosphorylates and inhibits the glu-tRNA synthase, inhibiting protein synthesis (72). Inhibition of protein synthesis is an effective means to produce multidrug tolerance. For example, bacteriostatic antibiotics erythromycin (73) and chloramphenicol (74) inhibit translation and lead to tolerance of other antibiotics, and this is why they are not used in combination with β-lactams. How exactly inhibition of translation causes tolerance is unclear, though it probably involves shutdown of other antibiotic targets by feedback inhibition. A deletion in *hipBA* has no phenotype in E. coli, but a gain-of-function *hipA7* allele sharply increases persister formation *in vitro* (75) and, importantly, in clinical isolates of patients with urinary tract infection (9). In a recent study, we measured ATP in individual persisters surviving treatment with ampicillin in a microfluidics device (28). Most persisters had low ATP levels, but in about 20%, ATP was similar to regular cells. Linking single-cell analysis of noise in particular genes to antibiotic tolerance will reveal additional mechanisms of persister formation.

## MATERIALS AND METHODS

### Bacterial strains, culture conditions, and strain construction.

E. coli strains were grown in either LB broth (Fisher Scientific, USA), morpholine propanesulfonic acid minimal medium (76) supplemented with 0.4% glucose, 0.4% sodium succinate, or 0.4% sodium pyruvate, M9 minimal medium (Fisher Scientific, USA) supplemented with 1% lactose and thiamine, or LBB supplemented with 0.2% melibiose as indicated. Cultures were grown at 37°C with shaking at 220 rpm. Strains containing pBAD33-*melR* were grown in LBB supplemented with 0.2% melibiose and chloramphenicol at 30 μg/ml to maintain plasmids. *melR* expression was induced with 0.1% l-arabinose overnight. MG1655 was used for these studies, and single deletions of global regulators were transduced from the Keio collection (53). Deletions were confirmed by amplifying upstream and downstream of the gene of interest. Reporter fusions for *gltA*, *sucA*, *aceA*, *icd*, and *pgk* were transduced via P1 phage transduction from the mVenus collection (28).

### (i) Construction of *ihfA-mVenus*.

The *ihfA-mVenus* fusion was constructed as schematized previously (28). Briefly, the 5-amino-acid linker, mVenus fluorescence protein, and chloramphenicol cassette were amplified from *aceA-mVenus* (28) with the primers listed in Table S1 in the supplemental material and contain 50 bp of homology. The resulting PCR product was inserted into MG1655 using λ red recombination as described previously (62). The fusion was confirmed by amplifying upstream and downstream of the *ihfA* gene.

10.1128/mbio.03420-21.1 TABLE S1 Primers used in this study Table S1, PDF file, 0.03 MB. Copyright © 2022 Nicolau and Lewis. 2022 Nicolau and Lewis. https://creativecommons.org/licenses/by/4.0/ This content is distributed under the terms of the Creative Commons Attribution 4.0 International license.

### (ii) Construction of Δ*lacI*.

The kanamycin resistance cassette was amplified from the Keio collection with the primers listed in Table S1 and contains 50 bp of homology to the upstream and downstream regions of *lacI.* The amplified cassette was inserted into MG1655 using λ red recombination as described previously (62). The deletion was confirmed by amplifying upstream and downstream of the *lacI* gene.

### Persister assays.

Cultures were grown to stationary phase overnight in 2 ml of the medium indicated in a 14-ml capped culture tube (VWR International), and the starting CFU were plated. Cultures were challenged with ciprofloxacin at 10 μg/ml, gentamicin at 50 μg/ml, or ampicillin at 100 μg/ml. To enumerate survivors, 100 μl culture was taken, pelleted by centrifugation, washed with phosphate-buffered saline (PBS), serially diluted, and plated on LB agar plates. Plates were counted after 24 h of growth to enumerate survivors. Experiments were performed in biological triplicates.

### ATP quantification.

ATP levels were measured using the Promega BacTiter-Glo microbial cell viability assay according to the manufacturer’s instructions; 100 μl culture was used as a working volume for reading luminescence. Experiments were performed in biological triplicates.

### FACS analysis using mVenus reporters.

Fluorescence activated cell sorting was performed on a BD Aria II flow cytometer (BD Biosciences) with a 70-μm nozzle. For sorting experiments, reporters were grown to stationary phase in media containing various carbon sources and then challenged with ciprofloxacin at 10 μg/ml for 24 h before sorting. After 24 h, cultures were washed, diluted 1:100, and sonicated. The initial population of cells was gated by size using forward scatter (FSC) and side scatter (SSC) and then on the basis of green fluorescent protein (GFP) fluorescence (FITC-A) using the FACS Diva software. Gates were set to include the dimmest 5% and brightest 5% of the population as well as the bulk of the population. Cells from each population were then sorted onto LBA plates and incubated at 37°C for 24 h. Colony counts were normalized for expected colony counts by an untreated sort plate. Percent survival was calculated for dim, bright, and GFP-positive fractions. For analysis of TCA cycle gene fusions, the initial population of cells was gated by size using FSC and SSC. Fluorescence data from 100,000 events was collected and analyzed using FlowJo software.
