# Supplementary material for: The Role of Integration Host Factor in Escherichia coli Persister Formation
Source: mBio. 2022 Jan 4;13(1):e03420-21. doi: 10.1128/mbio.03420-21 (PMC8725577; doi:10.1128/mbio.03420-21)
Supplement: TABLE S1 [file mbio.03420-21-st001.pdf]

Purpose	Type	Sequence (5' to 3')
Construction of *ihfA*-*mVenus*	Forward	GGCAGAAGTTAAAAAGCCGGGTCGAAAACGCTTCGCCCAAAGACGAGACTAGTGCGGCCGCGGTGAG
	Reverse	GACAGTGAAAAGAAAAAAGGCCGCAGAGCGGCCTTTTTAGTTAGATCAGACGCTGGACTACCGAAGATTC
Verification of *mVenus* fusions	Forward	ACTAGTGCGGCCGCGGTGAGCAAGGGCGAGGAGC
	Reverse	CGCTGGACTACCGAAGATTC
Construction of *lacI* deletion	Forward	CGGTATGGCATGATAGCGCCCGGAAGAGAGTCAATTCAGGGTGGTGAATATGATTGAACAAGATGGATT
	Reverse	AAGCCTGGGGTGCCTAATGAGTGAGCTAACTCACATTAATTGCGTTGCGCTCAGAAGAACTCGTCAAGAA
Cloning of *melR* into pBAD33	Forward	AATTGGTACCCCAGGAAAGAGAGCCATCCATG
	Reverse	CCGGAAGCTTTTAGCCGGGAAACGTCTGGC
